# Passage of *Angiostrongylus cantonensis* through the trophic web: an experimental study on reptiles

**DOI:** 10.1017/S0031182025000034

**Published:** 2025-01

**Authors:** Lucia Anettová, Anna Šipková, Vivienne Velič, Jana Kačmaříková, Kristýna Javorská, Ladislav Novotný, Petr Cibulka, Martin Květoň, David Modrý

**Affiliations:** 1 Department of Veterinary Sciences, Czech University of Life Sciences, Prague, Czech Republic; 2 Department of Botany and Zoology, Masaryk University, Brno, Czech Republic; 3 Department of Pathology and Parasitology, University of Veterinary Sciences Brno, Brno, Czech Republic; 4 Department of Comparative Pathobiology, College of Veterinary Medicine, Purdue University, Lafayette, IN, USA; 5 Third Faculty of Medicine, Charles University, Prague, Czech Republic; 6 Clinical and Transplant Pathology Centre, Institute for Clinical and Experimental Medicine, Prague, Czech Republic; 7 Institute of Parasitology, Biology Center of Czech Academy of Sciences, Ceske Budejovice, Czech Republic

**Keywords:** *Angiostrongylus cantonensis*, intermediesis, paratenic host, rat lungworm, reptiles

## Abstract

The rat lungworm *Angiostrongylus cantonensis* is a zoonotic metastrongyloid nematode, currently considered an emerging pathogen approaching Europe. In tropics and subtropics, it is an important food-borne neurotropic parasite of medical and veterinary importance. Sources of infection for mammals and birds include gastropod intermediate hosts and poikilothermic vertebrates (paratenic hosts). To evaluate the relevance of reptiles in the rat lungworm circulation, we performed an experimental series focused on long-term survival of third stage larvae (L3) of *A. cantonensis* in reptiles and potential of saurians to serve as a source of infection for further hosts. Twenty leopard geckos (*Eublepharis macularius*) were infected with varying doses of L3 (100, 1000, 10 000 larvae per animal). Live L3 were collected from all infected geckos (mostly in musculature and liver) euthanized 1–6 months after the infection and were proven to be infective for Wistar rats (definitive hosts). Three sacrificed geckos were subsequently fed to three corn snakes (*Pantheropis guttatus*) to test hypothesis of L3 infectivity for predators positioned higher in the food chain. Snakes were euthanized 1 month post-infection and live L3 were detected predominantly in the intestinal wall. The animals remained clinically healthy throughout the study. No reptiles showed significant changes in haematological and biochemical blood parameters, though elevated CK and GLDH were observed in most geckos in the group receiving higher infectious dose. This study highlights the significant potential of reptiles to play a crucial role in the circulation of metastrongyloid nematodes in food web and in their transmission to humans.

## Graphical abstract

**Figure:**
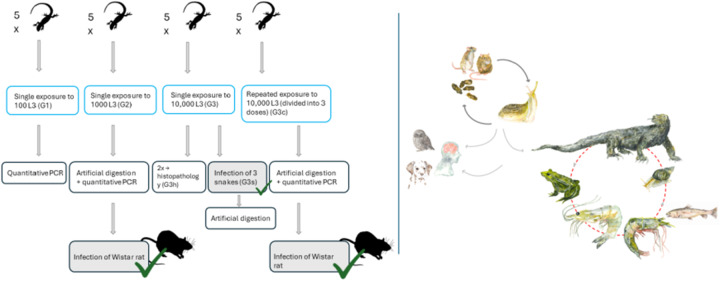

## Introduction

**Figure 1.**
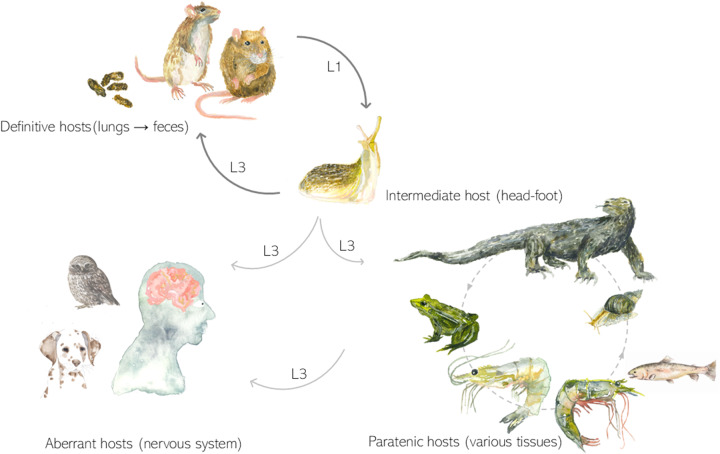
Complex life cycle of *A. cantonensis*. Rats serve as definitive hosts and get infected by third-stage larvae (L3) from gastropods. Adults develop in their pulmonary arteries. Eggs are shed in rats’ faeces. Gastropods are infected by first-stage larvae (L1) by consuming the rat faeces and L3 develops in their tissues. Many species of poikilotherms can serve as paratenic hosts and larvae can be transmitted among them as well as to the definitive or aberrant hosts. Aberrant hosts get infected by L3 (from gastropods, paratenic hosts or contaminated food or water) and can suffer from neurological disorder.

Heteroxenous life cycle involving gastropods as intermediate hosts is a predominant way of transmission in metastrogylid nematodes (Strongylida: Metastrongyloidea) affecting mammalian hosts (Anderson, 2000). However, paratenic hosts play a crucial role in the metastrongylids’ circulation, filling a trophic gap in their life cycle (Figure 1), given many carnivorous and rodent definitive hosts do not often consume gastropods deliberately (Odening, 1976; Bush, 2001). Almost every class of poikilothermic vertebrates has been mentioned as a potential reservoir of infective larvae of *Angiostrongylus cantonensis*, commonly known as the rat lungworm. This zoonotic nematode, classified within the family Angiostrongylidae, draws significant public attention due to its neuropathogenic impact on humans and other mammalian and avian hosts. In poikilotherms, which serve as paratenic hosts of the parasite, the infective third-stage larvae (L3) do not progress beyond their larval stage. Instead, they migrate to the viscera and skeletal muscles, where they accumulate and serve as a potential source of infection of vertebrates at higher trophic level (Turck et al., 2022). Conversely, in birds and mammals, these larvae become lodged within the central nervous system, leading to neurological disorders (Kim et al., 2002; Monks et al., 2005; Spratt, 2015; Delgado-Serra et al., 2022). The rat lungworm is known to have originated in Southeast Asia. Since then, it has spread into tropics and subtropics throughout the world, being considered as an emerging pathogen of global importance. It is known to adapt to new host spectra regarding its intermediate (and paratenic) hosts in non-indigenous areas of its distribution. This phenomenon has been repeatedlyobserved in isolated island ecosystems characterized by their unique endemic fauna (Martín-Carrillo et al., 2021; Jaume-Ramis et al., 2023).

Reptiles are known to serve as paratenic hosts of *A. cantonensis* and can be a source of infection for humans and animal when eaten raw or undercooked (Turck et al., 2022; Pandian et al., 2023). In Asia, yellow tree monitor lizards (*Varanus bengalensis*) are the most common reptile source of infective larvae for humans (Kanpittaya et al., 2000; Hidelaratchi et al., 2010; Yang et al., 2021). These lizards are considered a delicacy (typically in India and Sri Lanka) (Hidelaratchi et al., 2010) and, their meat or liver are used in traditional medicine, as believed to strengthen human power (Wangpan and Tang Jang, 2012). Reptiles, in particular geckos, were also reported as a suspected source of infection of captive birds of prey infected with the rat lungworm (Burns et al., 2014). Monitor lizards appear to be highly susceptible to infection with *A. cantonensis* (Radomyos et al., 1992), and therefore may play an important role in the parasite’s circulation. Other reptile species have also been found to naturally harbour *A. cantonensis* larvae. Recently, endemic lizards (*Gallotia galloti*) were confirmed as paratenic hosts of the nematode in the Canary Islands, a location near Europe (Anettová et al., 2022).

Experimental studies on reptiles as hosts of *A. cantonensis* are limited. Ash (1968) infected sea snakes (*Laticauda colubrina*), recovering infective larvae after 1–4 weeks mostly from somatic musculature. Radomyos et al. (1992) demonstrated *Varanus bengalensis* as a paratenic host, capable of harbouring infective larvae for up to a month. Subsequently, a survey in Thailand by Radomyos et al. (1994) found 21 out of 23 monitor lizards infected, primarily in the liver.

While the observation of wild animals provides essential data on the parasite’s distribution, insufficient information about the animals’ history complicates the evaluation of detailed infection dynamics and impact of infection on hosts’ fitness. Experiments in controlled environments can overcome these obstacles. Since it is already known that reptiles, especially saurians, can harbour infectious larvae of *A. cantonensis*, our study aimed to investigate more complex interactions. We conducted a series of experimental infections on model reptilian hosts (leopard geckos, *Eublepharis macularius*) and corn snakes (*Pantherophis guttatus*) to demonstrate how exposure to various doses of infective larvae influences the final number of larvae in the host’s tissues and to determine if the larvae can survive in reptiles for extended period of time. We also hypothesized that the L3 survive and accumulate in reptiles at higher trophic level (represented by snakes in our experiment) after predation on infected lizards, explaining high infection intensities in Asian monitor lizards. Additionally, cannibalism among conspecific *Gallotia* spp. can potentially lead to larval transmission from one lizard to another as well. This phenomenon of metastrongylid larvae transmission between 2 hosts on the same level has been described for *A. cantonensis* (Modrý et al., 2021) as well as other metastrongylids (Colella et al., 2015). Our final objective was to assess the impact of infection on the fitness of experimental lizards.

## Materials and methods

The experimental strain of *A. cantonensis* was obtained in 2021 in Tenerife, Spain and is maintained in laboratory conditions circulating between Wistar rats and experimental gastropods (*Lissachatina fulica* and *Veronicella cubensis*). The identity of the isolate was verified by examining the morphology of adult nematodes recovered from infected rats, and it was classified as part of the *A. cantonensis* clade 2 through cox1 sequencing (as described by Červená et al. (2019)). The live L3 were obtained by artificial digestion, using 0.3 g pepsin (3.4.23.1 (BRENDA, IUBMB)) and 100 mL 0.7% HCl on a magnetic stirrer set to 0.6 × ***g*** and 37°C (as in Modrý et al. (2021)) and larvae were collected either manually (G1 and G2) or using an aliquot of a volume containing specific number of L3 (G3). A scheme depicting the experimental groups is shown in detail in Supplementary Figure S1.

Leopard geckos were obtained from a private breeder as sub-adults, approximately 12 months old. They were allowed to acclimatize for 2–4 weeks and were kept in terraria in same-sex pairs. They were fed with crickets (commercially available) 3 times per week and mineral supplements were provided. Adult corn snakes of approximately 2 years of age were obtained from a private breeder as well. Snakes were let to acclimatize for 4 weeks and were fed every third week with commercially available sacrificed mice. During the first phase of the experiments, 10 leopard geckos were used to assess the dose needed for retrieving of L3 for infection of further hosts (definitive and paratenic). Five leopard geckos (females, group G1) were inoculated with a suspension of 100 L3 each, using orogastric tube (‘low dose’ group). Five male leopard geckos (G2) were inoculated with a higher dose of 1000 L3 each. The remaining 3 male geckos (G0) served as a control group to assess possible changes in clinical status and in haematological and biochemical parameters.

Animals in all 3 groups were euthanized 4 weeks after the infection, using dexmedetomidine (0.3 mg/kg) and ketamine (3 mg/kg) followed by intracardial application of pentobarbital (0.2 mL). Before the application of pentobarbital, blood was collected from heart for haematological and biochemical analyses. White blood cells, heterophils, lymphocytes, monocytes, eosinophiles and basophiles were counted, and following enzymes were quantified: ALT (alanine aminotransferase), AST (aspartate aminotransferase), ALP (alkaline phosphatase), GLDH (glutamate dehydrogenase), CK (creatine kinase) and TP (total protein) (in collaboration with Laboklin Czech). Due to small body size of the animals, it was not possible to take sufficient amount of blood for the analyses intravitally (i.e. prior to the infection). Clinical status of geckos and snakes was checked daily, and their food intake was documented. During necropsy, samples for histopathological examination (approx. 0.3 cm^3^) were taken from following tissues: liver, tail muscle, proximal hind leg muscles (i.e. mainly m. ambiens and m. flexor tibialis externus anterior, according to Zaaf et al. (1999)), heart, intestine and brain. After at least 24 h fixation in alcohol formalin glacial acetic fixative (AFA), tissue samples from G2 and G3h groups were embedded in paraffin and 6 μm sections were cut transversally in 4 planes, stained with haematoxylin–eosin (H&E) for histology in St. Anne’s University Hospital, Brno. Additionally, a small piece of each tissue (approx. 30 μg) was taken for a qPCR analysis (quantification for a comparison among tissues). All the remaining tissues were artificially digested and live L3 were collected for bioassay on 2 Wistar rats (40 L3 for each, infected under sedation using dexmedetomidine 0.2 mg/kg and ketamine 3 mg/kg). In the second part of the experiment, 10 geckos (G3, divided into 3 subgroups, G3s, G3h and G3c) were infected with a high dose of up to 10 000 L3 each. These doses were divided into 3 for each animal and were applied for 3 consecutive days (using the same method as described above) to avoid regurgitation caused by an excessive amount of a liquid applied. Group G3s contained 3 geckos (1 male and 2 females) which were intended to be ingested by snakes. Group G3h with 2 female geckos was used primarily for histopathological examination (as described for the previous groups), and the remainders of the bodies were artificially digested as described in G2. Group G3c (5 geckos, 4 females and 1 male) were infected continually, 3 times (with an interval of 1 week) with approximately 3000 L3 each time. This group was intended to assess the long-term infection (6 months). After 4 weeks post-infection, animals from G3s and G3h were sacrificed. In animals from G3s, which were intended for a consumption by snakes, dexmedetomidine (0.3 mg/kg, i.m.) + ketamine (1 mg/kg) was applied, followed by rapid destruction of brain (as pentobarbital could not be used due to further consumption by a snake). Animals from the group G3h were euthanized following the same protocol as in G1 and G2 (dexmedetomidine (0.3 mg/kg) and ketamine (3 mg/kg) followed by intracardial application of pentobarbital, 0.2 ml pro toto). The animals from the group G3c were euthanized 6 months after the end of infection period and processed in the same way as G1 and G2, except for the histopathological examination, which was not performed in this group. Larvae collected during microscopic examination after artificial digestion of tissues were used for a bioassay on Wistar rats. Two rats were each infected with 40 L3 larvae collected from geckos euthanized 1 month post-infection. One Wistar rat was infected with 40 L3 larvae collected from geckos euthanized 6 months post-infection. The rat faeces were examined 45 days post-infection to confirm the shedding of L1 larvae of *A. cantonensis*.

### qPCR analysis

Samples from the organs (liver, intestine, heart and brain) and muscles from experimental groups G1, G2, G3h and G3c were weighed and up to 50 mg of tissue was cut into small pieces and used for the DNA extraction with Zybio (EXM3000) extraction kit with modification optimized for L3 of *A. cantonensis*, when the pre-lyse phase was extended overnight. Samples were examined for the presence of *A. cantonensis* DNA by a species-specific qPCR assay (Sears et al., 2021). The assay was performed in a 20 μL reaction using 6.2 μL of PCR water, 10 μL of 2 × MasterMix (IDT Prime time gene expression master), 0.2 μL of 10 μM probe (PrimeTime Eco Probe 5ʹ 6-FAM/ZEN/3ʹ IBFQ, /56-FAM/ACA TGA AAC/ZEN/ACC TCA AAT GTG CTTCGA/3IABkFQ/), 0.8 μL of each 10 μM primer (forward: AAA CTG TTG CTT TCG AAG CTA TG and reverse: GCG CAA ATC TGA CGT TCT TG) and 2 μL of DNA template. Thermocycling (40 cycles) was made with the following cycling conditions: 95°C for 20 sec followed by 40°C for 1 sec and 60°C for 20 sec. As a positive control, DNA from a single L3 of *A. cantonensis* extracted by the same method as samples and diluted 10× were used. We quantified the larval amount per gram of tissue by using a standard curve. The standard curve was designed based on the extracted DNA of a single *A. cantonensis* L3 diluted 1 ×, 10 ×, 100 × and 1000 ×. For all the qPCR runs, 100× dilution was used as a standard and the standard curve was applied. The actual DNA amount per gram of tissue was recounted for the weight of the tissue used for the DNA extraction and the data on the DNA amount were used for a comparison of the infection intensity of the muscles and viscera.

### Statistical analysis

To assess the normality of the data, Shapiro–Wilk and Levene tests were performed to decide whether to use Kruskal–Wallis or ANOVA. Kruskal–Wallis test (*R*) was used to compare the infection intensities represented by calculated concentrations of the DNA among different organs (liver, muscle, gastrointestinal tract, i.e. GIT, heart and brain) in groups G1, G2, G3c and G3h. Dunn’s test was used for post hoc pairwise comparisons generated by Kruskal–Wallis test. All the blood parameters, haematological as well as biochemical in groups G0 (negative control), G1 (low dose, 100 L3) and G2 (intermediate dose, 1000 L3) were analysed individually and subsequently compared by ANOVA (*R*). In snakes, we compared the same blood parameters as in geckos before and after the infection by *t*-test (*R*).

### Histopathological analysis

After at least 24 h fixation in AFA, tissue samples from G3h group and all the snake tissues were embedded in paraffin and 6 μm sections were cut transversally in 4 planes, stained with H&E for histology in St. Anne’s University Hospital, Brno. Stained slides were examined under a light microscope to assess the distribution and morphology of the larvae as well as the tissue response. Additionally, the Chromotrope 2R staining was performed following a modification of the original method described by Lendrum et al. (1962). Briefly, tissue sections (4 μm) were deparaffinized and hydrated through graded alcohols to water. The slides were then stained in a solution containing 1% Chromotrope 2R and 0.5% phenol in distilled water at 56°C for 30 min. Slides were counterstained with Mayer’s haematoxylin for nuclear detail, dehydrated, cleared and mounted. This method selectively stains eosinophilic granules bright red while providing excellent contrast for nuclei and other tissue elements. The Sirius Red staining was carried out according to the method described by Llewellyn (1970). Tissue sections were deparaffinized and rehydrated through graded alcohols. Nuclei were stained using a Weigert’s iron haematoxylin. The slides were then stained in an alkaline Sirius Red solution (0.5% Sirius Red F3B in a solution adjusted to pH 8–9) for 2 h at room temperature. After staining, sections were rinsed thoroughly with distilled water, dehydrated, cleared and mounted. This method stains amyloid, eosinophil granules and Paneth cells red, providing clear visualization against a contrasting background. Special stains were carried out at the Institute of Clinical and Experimental Medicine in Prague.

## Results

### L3 survived and remained infective in all the tested animals

Tissues of all individuals from G2, G3h and G3c were artificially digested following dissection and sample collection for the qPCR and histopathological examination. In the group G2, 4 out of 5 individuals contained tens of live L3 mostly in their skeletal muscles of the body, less in tail muscle and only a small fraction in viscera, mostly in liver. In the group G3h (2 individuals euthanized 1 month post-infection), hundreds of live L3 were present in body and tail musculature and only a small fraction (13 in one individual and 0 in the other one) in liver. In these animals, approximately half of each organ was artificially digested, and the other half was used for the histopathological examination, which means the total number of L3 in each tissue would be approximately twice as large as in the digested part. In the G3c group, where animals were euthanized 6 months post-infection, live L3 were still found, predominantly in the musculature. However, their numbers were lower compared to those observed in animals euthanized 1 month post-infection. The animals from G3s were used for the snake infection. Two snakes ingested sacrificed geckos from G3s group spontaneously and one snake was force-fed. Live L3 were found in all snakes (euthanized 1 month post-infection), predominantly in the GIT wall, less in liver and somatic musculature.

**Figure 2.**
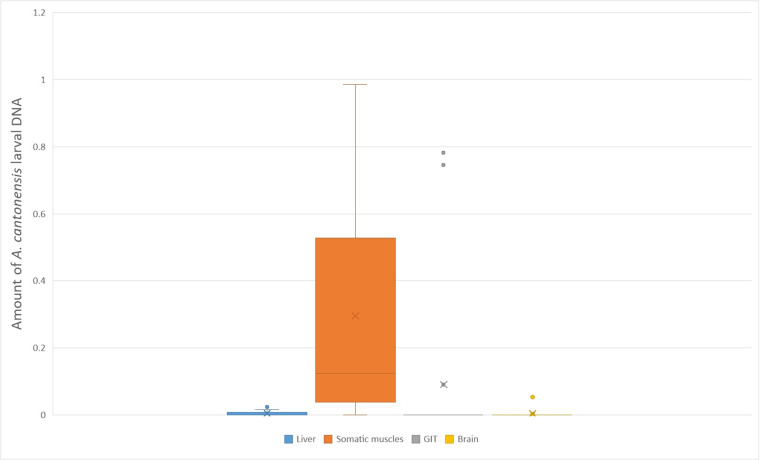
A boxplot graph depicting larval burden with *A. cantonensis* L3 in various tissues of experimentally infected leopard geckos, as counted from the quantitative PCR analysis (Supplementary Table S1). Somatic musculature is the most intensively infected tissue. Negative tissues were excluded from the graph. The X marks the average value, the median is indicated by a line across the box. The whiskers on box plots show the ranges of Q1 and Q4 up to the most extreme data points.

All the Wistar rats inoculated with L3 from geckos (1 as well as 6 months post-infection) were shedding L1 45 dpi, thereby confirming the viability of the L3 larvae and their infectivity for the definitive host. Detailed counts of larvae retrieved from each individual are provided in Table 1, while a graph illustrating the larval distribution is shown in Figure 2.

**Table 1.** Numbers of collected third stage larvae (L3) from the tissues of leopard geckos and corn snakes infected with *A. cantonensis*. Only liver of all the viscera contained live larvae after artificial digestion. Corn snakes (*Pantheropis guttatus*) were infected via intermediesis, i.e. each corn snake ingested a gecko infected with approx. 10 000 L3 1 month post-infection. Based on another group infected the same way with the same amount of L3, we assume each snake ingested a gecko containing approx. 500 L3

Individual	L3 in body muscle	L3 in tail muscle	L3 in liver
G2-1	0	0	0
G2-2	12	1	1
G2-3	36	8	1
G2-4	33	16	4
G2-5	32	8	1
G3h-1	242	78	0
G3h-2	208	62	13
G3c-1	57	30	0
G3c-2	3	22	1
G3c-3	14	6	0
G3c-4	4	16	0
G3c-5	88	10	2

### Muscles were the most intensively infected tissues in geckos

**Figure 3.**
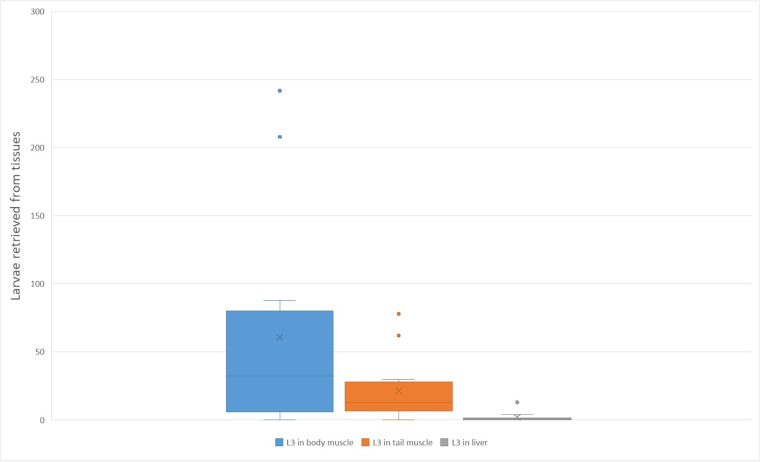
A boxplot graph depicting L3 of *A. cantonensis* from various tissues of experimentally infected leopard geckos (Table 1). Somatic musculature is the most intensively infected tissue. The X marks the average value, the median is indicated by a line across the box. The whiskers on box plots show the ranges of Q1 and Q4 up to the most extreme data points.

For some of the tested groups, the data on infection intensities were insufficient to meet the assumptions of the Shapiro–Wilk test. Given the distributional characteristics of the data and the violation of normality assumptions, we proceeded with the Kruskal–Wallis test, which does not assume normality. A Kruskal–Wallis test showed a statistically significant difference in infection intensities across the leopard geckos’ tissues, *χ*^2^(4) = 10.318, *p* = 0.0354. Post hoc pairwise comparisons using Dunn’s test with Bonferroni correction revealed significant differences between muscles and other tissues (*p* < 0.05), indicating higher infection intensities in muscles compared to other tissues in leopard geckos. A graph illustrating the distribution of tissues with varying DNA amounts of *A. cantonensis* is shown in Figure 3.

### Histopathological analysis

**Figure 4.**
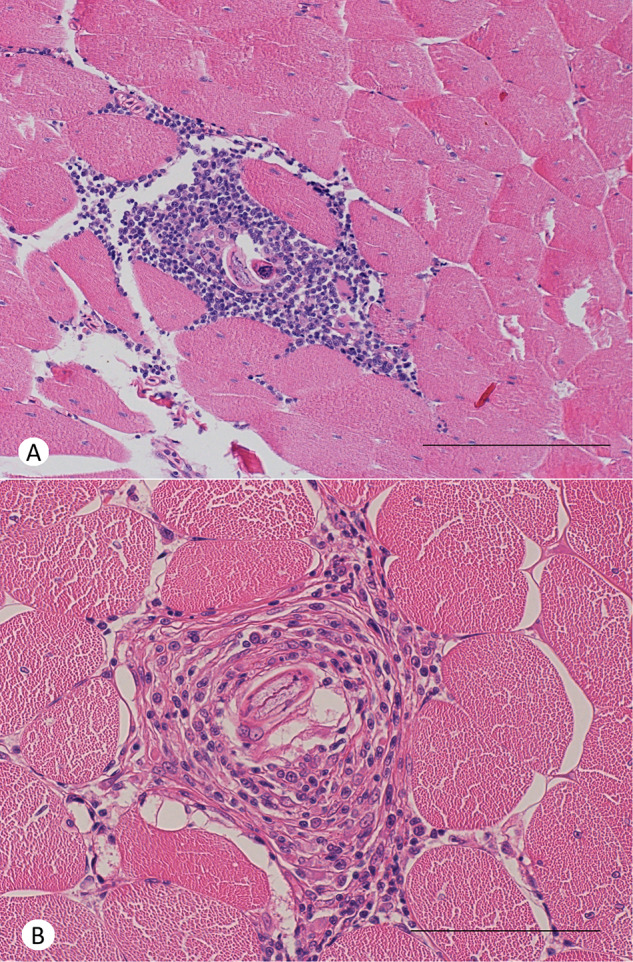
Histological section of skeletal muscle of a leopard gecko (*Eublepharis macularius*) 30 days post-infection by *A. cantonensis*. In both cases, endomysium of a skeletal muscle is focally expanded by inflammatory infiltrate around larva of *A. cantonensis*. (A) The inflammation is composed mainly of macrophages with moderate numbers of small lymphocytes. Scale bar = 200 μm. (B) The inflammation is composed mainly of macrophages with occasional small lymphocytes. Haematoxylin–eosin stain. Scale bar = 100 μm.

**Figure 5.**
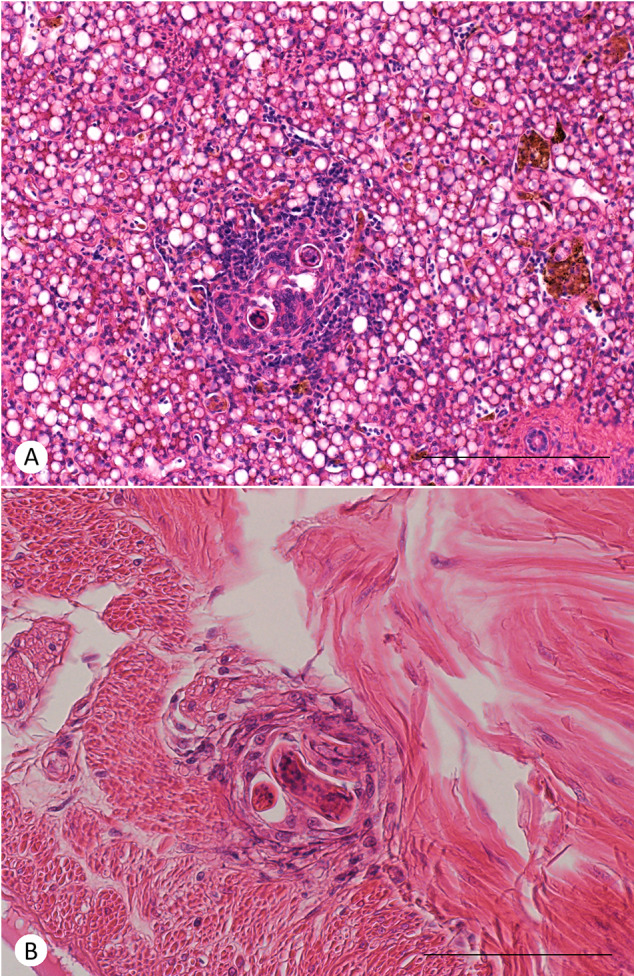
Histological section of viscera of a corn snake (*Pantheropis guttatus*) 27 days post-infection by *A. cantonensis* (snakes were infected by intermediesis, i.e. ingestion of an infected leopard gecko). (A) Hepatic parenchyma is focally expanded by inflammatory infiltrate around larvae of *A. cantonensis*. The inflammation is composed mainly of macrophages with moderate to large numbers of small lymphocytes at the periphery. The cytoplasm of hepatocytes is expanded by large lipid vacuoles (lipidosis). Scale bar = 200 μm. (B) Subserosal tissue of the stomach at the transition to muscularis externa is expanded by focal accumulation of macrophages around larvae of *A. cantonensis*. Haematoxylin–eosin stain. Scale bar = 100 μm.

**Figure 6.**
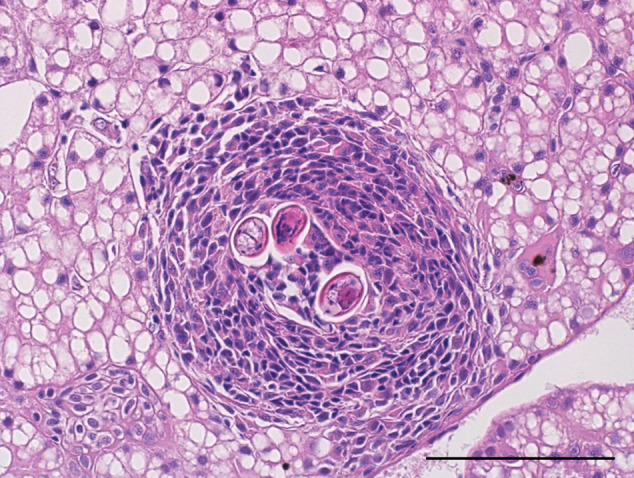
Histological section of a liver of a leopard gecko (*Eublepharis macularius*) 30 days post-infection by *A. cantonensis*. Hepatic parenchyma is focally expanded by inflammatory infiltrate around larvae of *A. cantonensis*. The cytoplasm of hepatocytes is expanded by large lipid vacuoles (lipidosis). The inflammation is composed mainly of macrophages with occasional small lymphocytes. Haematoxylin–eosin stain. Scale bar = 100 μm.

The larvae of *A. cantonensis* were observed in the endomysium of skeletal muscle tissue of geckos (Figure 4) and in the liver of geckos (Figure 6) and liver and stomach wall of a corn snake (Figure 5). The tissue inflammatory reaction was mild to moderate and morphologically similar in both species. The inflammatory infiltrate was composed mainly of macrophages and small lymphocytes. No obvious eosinophils were observed in H&E stain or in Sirius Red and Chromotrope 2R stain (Supplementary Figures S2 and S3). There is an increased accumulation of lipids in the cytoplasm of hepatocytes.

### Infection of paratenic hosts did not lead to clinical symptoms

None of the examined individuals, including both geckos and snakes, showed any clinical deterioration in their health status. However, we observed elevated levels of CK and GLDH, exceeding the reference range, in 3 out of 5 individuals in the G2 group (1000 L3 dose). These findings suggest potential indications of muscle damage (CK) and liver stress or damage (GLDH). In haematological parameters, we documented elevated monocytes in 2 individuals out of 5 in G2 and none in G1 (100 L3 dose) and G0 (negative control) eosinophils were elevated in all individuals throughout all tested groups. However, statistical analysis (ANOVA) of the blood parameters did not show any significant difference among the negative control, G1 (100 L3 dose) and G2 (1000 L3 dose); *p* > 0.05. Tested parameters and their values (as well as the used reference ranges) can be found in a Supplementary Table S1.

## Discussion

Complexity of the *A. cantonensis* life cycle apparently shows the importance of the parasite transmission within food webs. In this study, we aimed to provide more precise data on the transmission of *A. cantonensis* between 2 distinct groups of reptiles. We investigated the potential of geckos (or other small saurians) to serve as a source of infection for mammalian hosts. Additionally, we assessed the impact of the nematode on the fitness of these paratenic hosts. Our findings are in general consistent with the previous studies by Radomyos et al. (1992) and Ash (1968), however Radomyos et al. (1992) confirmed L3 presence predominantly in liver and in our case, somatic musculature was the most intensively infected tissue. It is worth noting that examining the whole musculature of animals as large as monitor lizards can lead to omitting the presence of small nematode larvae, therefore we used relatively small sub-adult leopard geckos to enable examining whole bodies.

Our study provides new insight into the pathophysiology of the nematode’s infection in studied reptile species including histopathological examination, as well as evaluating the larvae infectivity after a long-term survival in geckos. Additionally, this is a first report of intermediesis (transmission of L3 between hosts of the same level (Colella et al., 2015; Modrý et al., 2021)) between paratenic hosts in case of *A. cantonensis*. Larvae of the parasite were able to survive in leopard geckos for as long as 6 months, even though their numbers dropped over time. Furthermore, these larvae remained infective for the definitive host.

Snakes were infected with geckos which were inoculated with approx. 10 000 L3. Since whole bodies of geckos were fed to snakes, we were not able to evaluate the number of larvae in G3s, however based on the results from artificial digestion of G3h group where approximately half of each tissue was examined, we presume each snake ingested a gecko containing hundreds of L3 (approx. 500). The most significant difference between snakes and geckos were in the localization of larvae, while in geckos, most larvae were found in body musculature and significantly less in viscera (i.e. mostly in liver based on artificial digestion and qPCR analysis), in snakes, most larvae were found in the intestinal wall. The larvae localization in snakes in our study resembles the situation in crustaceans and fish, where larvae do not migrate as they do in lizards (Wallace et al., 1966; Wallace and Rosen, 1967). However, the larval survival in snakes is prolonged.

Neither geckos nor snakes did show any changes in their clinical status and blood parameters before and after the infection. Histopathological examination revealed that the arrested larvae and their migration induced lymphohistiocytic inflammation (early stage of granulomatous inflammation). However, immunological responses leading to these reactions do not appear to alter haematological parameters or affect the clinical status of the individuals. While we anticipated a notable increase in biochemical markers associated with larval migration in organs such as the liver and muscles (ALT, AST, GLDH and CK) following infection, our statistical analysis did not yield significant results. However, it’s noteworthy that in the majority of individuals infected with 1000 L3, levels of CK and GLDH, typically indicative of muscle and liver damage respectively, were higher than the reference range and this elevation was not reported in the low dose group and the negative control. Furthermore, it’s important to acknowledge the potential for subclinical impacts on the health of reptiles, even in the absence of significantly elevated blood markers. In this case, the presence of larvae in the muscles and liver likely triggered at least a minor reaction, although the effect on the biochemical parameters remained statistically insignificant. In general, studies on the host–parasite interaction between metastrongylid nematodes, i.e. larvae and their paratenic hosts are lacking, however granuloma formation (similar to aggregations of inflammatory cells in this study) was previously suggested to provide an effective evasion mechanism in *A. cantonensis* infected giant African snail *L. fulica* (Lopes-Torres et al., 2024). Not surprisingly, all the larvae in our study found in the histopathological sections were encapsulated inside a granuloma which seems to provide a barrier between a host and a parasite without causing a serious harm even with a quite high parasite load (Brockelman et al., 1976). The observed liver lipidosis is likely physiological, as certain poikilothermic animals, including reptiles, tend to accumulate lipids in the liver depending on the season (e.g. estivation or dormancy) (Price, 2016).

As previously mentioned, reptiles are part of the human diet in several Asian countries where *A. cantonensis* originates, and cases of eosinophilic meningitis resulting from their consumption have been reported. The parasite is now spreading to new areas, including closer to continental Europe, where lizard consumption is not common. Nevertheless, lizards and snakes remain a potential risk for neuroangiostrongyliasis in wildlife and contribute to the further spread of the parasite in regions where rats prey on lizards. We used doses of 100, 1000 and 10 000 L3 larvae to represent realistic scenarios for a lizard to harbour, considering that a single small snail can easily contain hundreds to thousands of L3 larvae (Kim et al., 2014; Rollins et al., 2021). In all experimental groups infected with various larval doses, only a fraction of L3 larvae survived in the reptile tissues. However, these doses would be still sufficient to cause serious harm to animals that prey on them in each ecosystem. Some larvae may have exited the GIT without leaving the lumen, entering the intestinal wall and migrating to the tissues. Based on our results and previous studies (Radomyos et al., 1992), saurians meet all the attributes of true paratenic hosts, as they can harbour live larvae for extended periods, unlike crustaceans or fish, which show only limited larval migration and only short period survival of L3 (Wallace et al., 1966; Wallace and Rosen, 1967). Lizards and geckos themselves are prey for numerous higher trophic level predators, such as birds of prey, snakes and mammals. This positions them as crucial intermediates, transferring energy and nutrients up the food chain (Bels et al., 2019). Considering the role lizards play in the food chain and their serving as paratenic hosts, we would like the underline how they can facilitate the persistence and spread of *A. cantonensis* within ecosystems.

The life cycle of *A. cantonensis* appears to be highly opportunistic, given the parasite’s ability to infect a wide variety of hosts and utilize numerous routes of infection. Additionally, *A. cantonensis*, with rats as definitive hosts and a broad range of gastropods as intermediate hosts, serves as a suitable laboratory model for studying similar metastrongylid nematodes’ circulation routes, due to its relative ease of maintenance in laboratory conditions.

## Supporting information

Anettov et al. supplementary material 1 Anettov et al. supplementary material

Anettov et al. supplementary material 2 Anettov et al. supplementary material

Anettov et al. supplementary material 3 Anettov et al. supplementary material

## Supplementary material

The supplementary material for this article can be found at https://doi.org/10.1017/S0031182025000034.
