# Supplementary figures and images for: Passage of Angiostrongylus cantonensis through the trophic web: an experimental study on reptiles
Source: Parasitology. 2025 Jan 22;152(1):115–22. doi: 10.1017/S0031182025000034 (PMC12088917; doi:10.1017/S0031182025000034)

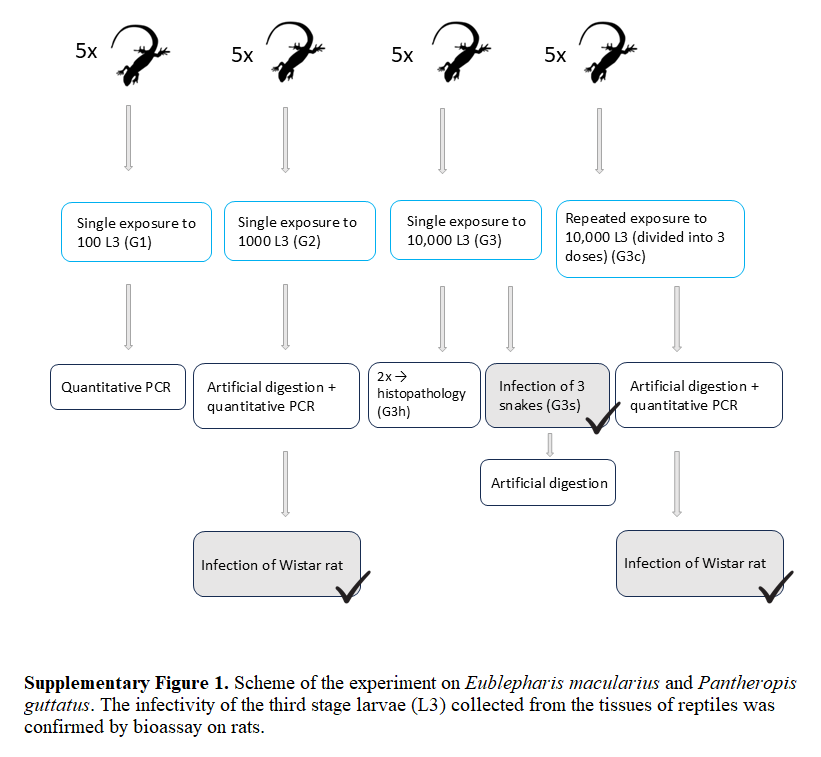

Supplement: Anettov et al. supplementary material 1 — Anettov et al. supplementary material [file S0031182025000034sup001.tif]

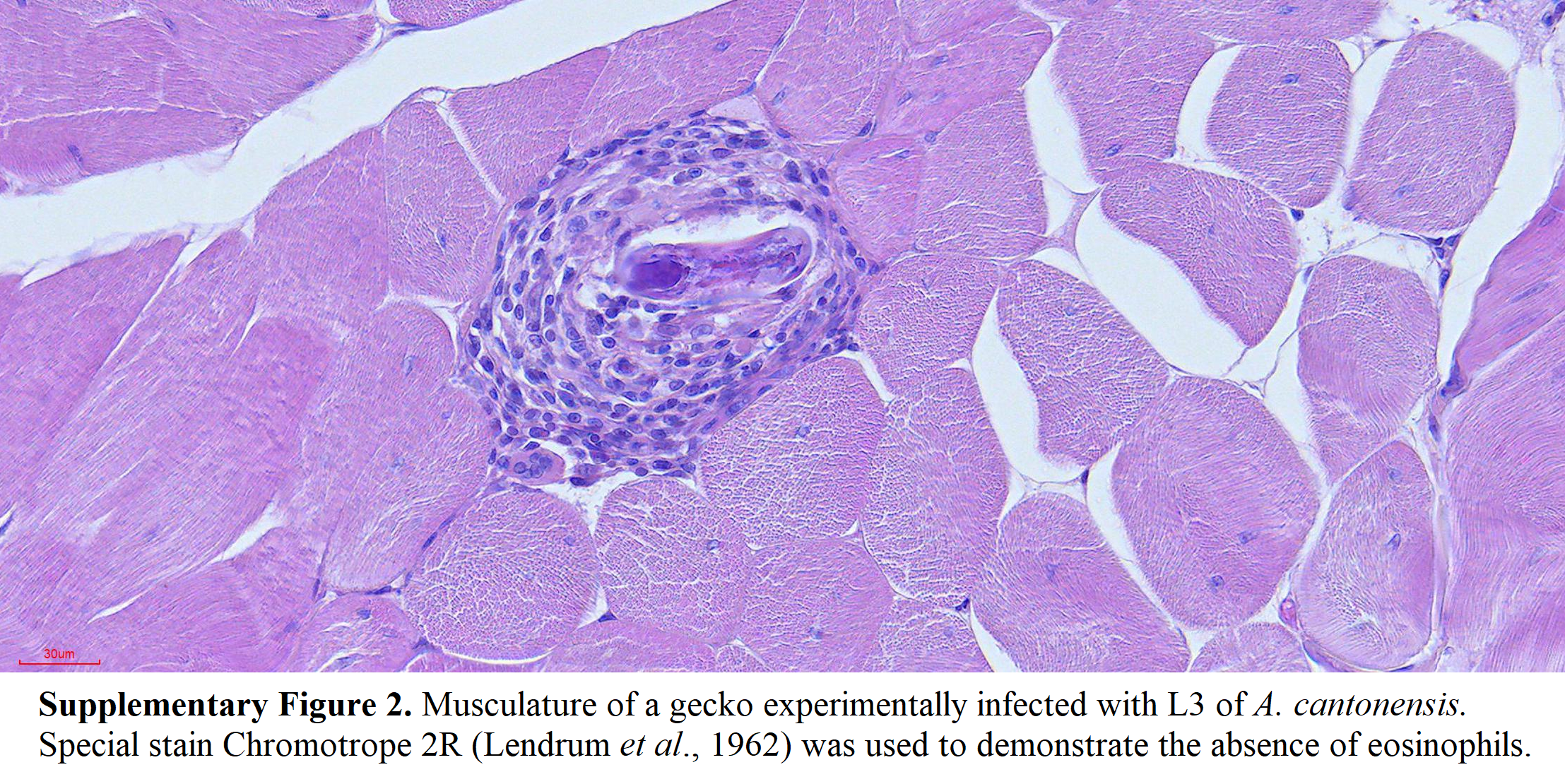

Supplement: Anettov et al. supplementary material 2 — Anettov et al. supplementary material [file S0031182025000034sup002.tif]

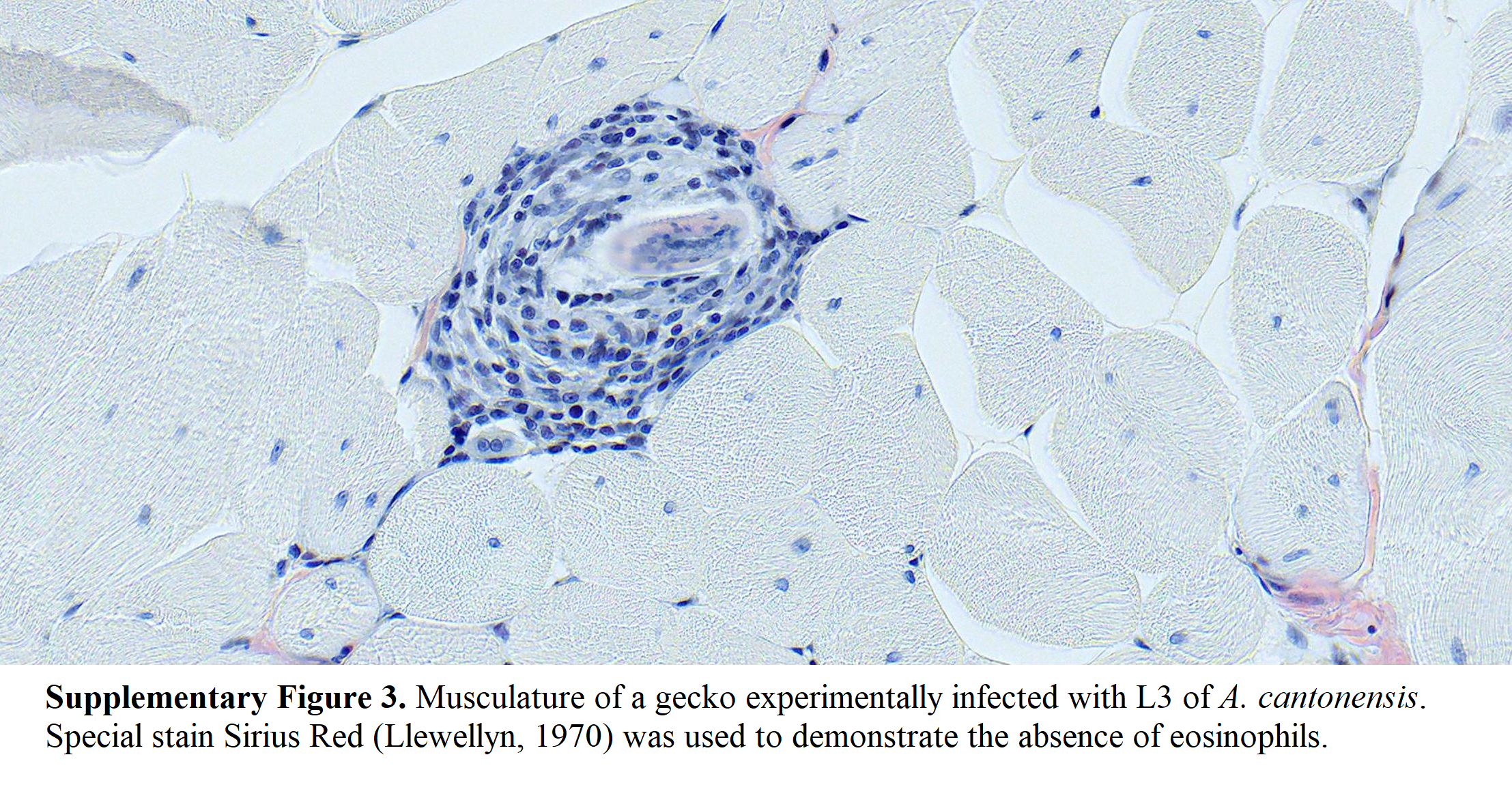

Supplement: Anettov et al. supplementary material 3 — Anettov et al. supplementary material [file S0031182025000034sup003.tif]
